# Phenotypic Assessment of Probiotic and Bacteriocinogenic Efficacy of Indigenous LAB Strains from Human Breast Milk

**DOI:** 10.3390/cimb44020051

**Published:** 2022-02-01

**Authors:** Senbagam Duraisamy, Fazal Husain, Senthilkumar Balakrishnan, Aswathy Sathyan, Prabhu Subramani, Prahalathan Chidambaram, Selvaraj Arokiyaraj, Wahidah H. Al-Qahtani, Jothiramalingam Rajabathar, Anbarasu Kumarasamy

**Affiliations:** 1 Microbial Biotechnology Laboratory, Department of Marine Biotechnology, Bharathidasan University, Tiruchirappalli 620 024, India; nevithasenthil@gmail.com (S.D.); fazal.husain.st@gmail.com (F.H.); aswathysathyan18.11@gmail.com (A.S.); 2 Department of Medical and Molecular Microbiology, Microtech Laboratories, Attur 636 102, India; nbsenthilkumar@gmail.com; 3 Department of Biochemistry, Bharathidasan University, Tiruchirappalli 620 024, India; sprabhubiochemistry@gmail.com (P.S.); prahalath@gmail.com (P.C.); 4 Department of Food Science and Biotechnology, Sejong University, Gwangjin-gu, Seoul 05006, Korea; arokiyaraj16@sejong.ac.kr; 5 Department of Food Science and Nutrition, College of Food & Agricultural Sciences, King Saud University, Riyadh 11451, Saudi Arabia; wahida@ksu.edu.sa; 6 Chemistry Department, College of Science, King Saud University, Riyadh 11451, Saudi Arabia; joeyashu77@gmail.com

**Keywords:** breast milk, lactic acid bacteria, probiotics, antimicrobial activity, bacteriocin

## Abstract

Breast milk is the combination of bioactive compounds and microflora that promote newborn’s proper growth, gut flora, and immunity. Thus, it is always considered the perfect food for newborns. Amongst their bioactives, probiotic communities—especially lactic acid bacteria (LAB)—are characterized from breast milk over the first month of parturition. In this study, seven LAB were characterized phenotypically and genotypically as *Levilactobacillus brevis* BDUMBT08 (MT673657), *L. gastricus* BDUMBT09 (MT774596), *L. paracasei* BDUMBT10 (MT775430), *L. brevis* BDUMBT11 (MW785062), *L. casei* BDUMBT12 (MW785063), *L. casei* BDUMBT13 (MW785178), and *Brevibacillus brevis* M2403 (MK371781) from human breast milk. Their tolerance to lysozyme, acid, bile, gastric juice, pancreatic juice, and NaCl and potential for mucoadhesion, auto-aggregation, and co-aggregation with pathogens are of great prominence in forecasting their gut colonizing ability. They proved their safety aspects as they were negative for virulence determinants such as hemolysis and biofilm production. Antibiogram of LAB showed their sensitivity to more than 90% of the antibiotics tested. Amongst seven LAB, three isolates (*L. brevis* BDUMBT08 and BDUMBT11, and *L. gatricus* BDUMBT09) proved their bacteriocin producing propensity. Although the seven LAB isolates differed in their behavior, their substantial probiotic properties with safety could be taken as promising probiotics for further studies to prove their in vivo effects, such as health benefits, in humans.

## 1. Introduction

Breast milk is the natural and safest biological fluid for newborns as it completely fulfills the nutritional and defense requirements. The health-promoting effect of breast milk, such as buildup of defense against various infectious diseases and boosting up the immune system, is probably due to the combined action of bioactives such as maternal immunoglobulins, immunocompetent cells, antimicrobial proteins (lactoferrin, CD14, and lysozyme), regulatory cytokines, and human milk oligosaccharides. Furthermore, breast milk is also a source of bacterial community that only establishes the infant’s gut flora and modulates their innate immunity [1,2]. Although the breast milk microbiome is diverse in nature, some factors—such as breastfeeding practices, behaviors, maternal factors, milk components, diet, delivery mode, and gestational age—are majorly influencing the diversity [3]. Furthermore, breast milk could be the most substantial source of potential probiotics since the predominant bacterial species include Bifidobacterial, Lactococcus, Lactobacilli, Enterococci, Streptococci, and Micrococci [4].

Probiotics are live microorganisms, conferring health benefits to the host when administered an adequate amount [5] by improving gut microbial equilibrium and immune function [6]. The use of probiotics has constantly been increased as scientific studies still continue to prove their beneficial efficiencies and functionalities on human health, especially to treat gastrointestinal tract (GIT) and vaginal infections [7]. Moreover, the effective therapeutic properties of probiotic preparations have been reported for traveler’s diarrhea [8], antibiotic-associated diarrhea [9], acute diarrhea [10], and inflammatory bowel disease [11]. These therapeutic roles are accomplished via various mechanisms such as competitive exclusion, modulation of the immune system, adherence to the epithelial cells, secretion of antimicrobial toxins, and enhancement of intestinal tight junction barriers [11,12]. Generally, probiotics have been isolated from various sources like vegetables, fruit juices, grains, honey comb, fermented dairy products, pickles, kafir, and miso [13]. Nevertheless, autochthonous probiotics—especially those isolated from the human gut, breast milk, and baby feces—have more advantages in their therapeutic application since these strains would easily colonize the gut after ingestion [14]. Moreover, breast milk probiotics would fulfill some criteria that are necessary for human probiotics, such as human origin, safe history, sustained intake by newborns, and easy adaptation to gastrointestinal conditions.

Several probiotics, such as *Lactobacillus crispatus* and *L. gasseri* [15], *L. plantarum* and *L. pentosus* [16], *L. rhamnosus* SHA113 [17], and *L. fermentum* [18] have been characterized from human milk and proved their efficiency to grow under gastrointestinal conditions. Nevertheless, the authorized and commercialized probiotics are very few in the market due to the need for extensive research to get approval. The National Health Commission of the People’s Republic of China recommended the breast milk probiotic, *Limosilactobacillus fermentum* CECT5716 from a Spanish mother’s milk for use in infant formula [1,19]. Thus, more efforts are necessary to study probiotics of human origin for their benefits as the selected probiotic candidates would be capable of performing efficiently in the GIT. Moreover, potent probiotic selection has generally been based on their ability of in vitro tolerance of various physiological stresses such as lysozymes, acid, bile, and osmolarity. In this pipeline, this study is aimed to obtain various LAB strains from breast milk collected from different lactating mothers in and around Tiruchirappalli, Tamil Nadu, India to check their probiotic potential using a series of in vitro experiments and their biosafety features.

## 2. Materials and Methods

### 2.1. Sample Collection, Processing, and Ethical Statement

In this study, 8 healthy volunteer mothers in the stage of lactation at 2–6 months are included and mothers under antibiotic treatment and with maternal perinatal disputes are excluded from this study. The milk samples were collected between 1–30 days of postpartum. For milk collection, sterile gloves were worn, the areola and nipple of the concerned mother were wiped with soap water, water, and finally with autoclaved distilled water in order to avoid skin bacterial flora. The first few drops of milk was discarded and next about 500 µL was collected by applying slight pressure on their breast manually. The milk sample was cooled immediately by keeping at 4 °C until delivery to the laboratory. Milk diluted in 0.1% peptone was plated in de Man Rogosa-Sharp (MRS) agar by pour plate method and incubated at 37 °C for 48 h. Colonies selected were subcultured 3–4 times in order to obtain pure culture and routinely identified for Gram’s staining, endospore staining, catalase, and oxidase reaction for screening LAB.

All volunteer mothers were signed in informed consent prior to study enrollment. All experiments were performed after approval by the institutional ethics committee for human research of Bharathidasan University, Tiruchirappalli (DM/2016/101/60).

### 2.2. Antimicrobial Spectrum of Bacterial Isolates

The pure culture of each isolate was grown in MRS broth for 24 h and cell free supernatant (CFS) was obtained by centrifuging at 10,000 rpm, 4 °C, for 10 min. The collected CFS was checked for their antibacterial activity against *Enterococcus faecalis* MTCC 439, *Shigella flexneri* MTCC 1457, *Aeromonas hydrophila* MTCC 1739, *Escherichia coli* MTCC 40, *Pseudomonas aeruginosa* MTCC 4679, *Staphylococcus aureus* MTCC 737, *Klebsiella pneumonaie* MTCC 39, and *Proteus vulgaris* MTCC 426 received from Microbial Type Culture Collection (MTCC), Chandigarh, India and clinical pathogens such as *P. aeruginosa*, *K. pneumoniae*, *Acinetobacter baumannii,* methicillin resistant *S. aureus* (MRSA) received from Mahathma Gandhi Memorial Government Hospital, Tiruchirappalli, Tamil Nadu, India. The assay was performed by well diffusion method using 50 µL of each indicator bacteria (2.1 × 10^5^ CFU/mL).

### 2.3. Preliminary Identification of Bacterial Isolates

The isolates showing wide antibacterial activity were tested for their physiological characterization by IMViC, urease, nitrate reduction, starch hydrolysis, and carbohydrate fermentation (lactose, glucose, mannitol, fructose, sorbitol, maltose, galactose, mannose, and arabinose). Negative control was always maintained for all the physiological tests.

### 2.4. Assessment of Probiotic Properties

#### 2.4.1. Tolerance to Lysozyme, Acid, and Bile

The lysozyme resistance of the isolates was checked by following Yadav et al. [20]. Shortly, 2% PBS suspended cell pellet containing 2.2 × 10^8^ CFU/mL was inoculated into sterilized electrolytes solution containing 0.22 g/L of CaCl_2_, 6.2 g/L of NaCl, 2.2 g/L of KCl_2_, 1.2 g/L NaHCO_3_, and 100 mg/L of lysozyme (Hi media, Mumbai, India) and incubated at 37 °C for 3 h (with shaking at 100 rpm). The aliquots taken from each broth culture were diluted and plated in MRS agar to enumerate the number of CFU. The survival percentage was calculated as [17]

(1) $$\mathrm{Survival}\;\%=\frac{\;\mathrm{Log}\;N1}{\mathrm{Log}\;N0}\times 100\;$$

where Log N1 is log CFU/mL from lysozyme added broth culture. Log N0 is log CFU/mL from broth culture without lysozyme (control).

The acid resistance was assessed in MRS broth, preadjusted to pH 2, 3, 4, and 7 (control). Similarly, bile tolerance was conducted in MRS broth added with 0.5% and 1% of bile (sodium taurocholate, Sigma Aldrich, India) by following the abovementioned method.

#### 2.4.2. Gastrointestinal Digestion

The artificial gastric fluid (AGF) at pH 2.5 was prepared by dissolving pepsin (3 mg/mL) in 125 mM NaCl, 7 mM KCl, and 45 mM NaHCO_3_ [21]. Similarly, artificial intestinal fluid (AIF) at pH 8 was prepared by dissolving 0.5% bile salt and 0.1% pancreatin (1 mg/mL) in 25 mM NaCl. Both the solutions were inoculated with 2% inoculum size containing 2.4 × 10^8^ CFU/mL and incubated in a shaking incubator at 37 °C for 2 h. Control was maintained in MRS broth at pH 7. The viable count was done before and after incubation and the survival percentage was calculated as described in Formula (1).

#### 2.4.3. Tolerance to NaCl

The resistance to osmotic stress was evaluated by growing the isolates in MRS agar broth containing various concentrations of NaCl: 0.3 mol/L (2%), 0.69 mol/L (4%), 1 mol/L (6%), 1.36 mol/L (8%) [22]. The bacterial growth was determined after 24 h incubation by reading the absorbance at 590 nm. Control was maintained in MRS broth without NaCl.

#### 2.4.4. Surface Adherence, Auto-Aggregation, and Co-Aggregation Assay

The adherence of bacterial isolates to various hydrocarbons reveals the physicochemical properties of the cell surface. This assay was performed using xylene, apolar solvent, ethyl acetate, monopolar and basic solvent and chloroform, and monopolar and acidic solvent. Auto-aggregation was performed by Kos et al. [23] and their percentage was calculated as

(2) $$\mathrm{Auto}-\mathrm{aggregation}\;\%=1-\frac{\mathrm{At}}{A0}\;\times 100$$

where At is the absorbance at different time points (1, 2, 3, 4, 5 h) and A0 is the absorbance at time 0 h.

Similarly, co-aggregation of probiotic isolates with pathogenic bacteria (*S*. *flexneri* and *E. fecalis*) was determined as described by Handley et al. [24] and the percentage was calculated as

(3) $$\%\;\mathrm{of}\;\mathrm{Co}-\mathrm{aggregation}\;=\left[\left(A1+A2\right)-\frac{2\left(\mathrm{Amix}\right)}{A1+A2}\times 100\right]$$

#### 2.4.5. Mucin Adherence

This assay was performed using gastric mucin, Type III from porcine (Sigma Aldrich) as the matrix. About 100 µL of mucin solution (10 mg/mL) in PBS was immobilized on the wells by incubating at 4 °C for 18 h. After washing with 200 µL of PBS, the wells were added with 100 µL bovine serum albumin (BSA) (20 g/L) (Sigma-Aldrich) in order to saturate the immobilized mucin and incubated at 4 °C for 2 h. The unbound BSA was removed by continuous washing with PBS (3 times). Then, 100 µL of each bacterial suspension containing 2.1 × 10^8^ CFU/mL was added to each well and incubated at 37 °C for 2 h and washed 4–5 times with PBS to remove unbound bacteria. Finally, 200 µL of 0.5% (*v*/*v*) Triton X-100 was added and kept at room temperature for 1 h to detach the mucin adhered bacterial cells from the well. The content of each well was thoroughly mixed by repeated pipetting, and 100 µL of the resulting suspension was plated to enumerate the CFU/well [16]. The percentage of mucin adherence was calculated by the formula

(4) $$\%\;\mathrm{of}\;\mathrm{mucin}\;\mathrm{adherence}=\frac{\log\;\mathrm{CFU}\;\mathrm{formed}\;\mathrm{after}\;\mathrm{adhered}\;}{\log\;\mathrm{CFU}\;\mathrm{formed}\;\mathrm{before}\;\mathrm{adhered}\;}\times 100$$

#### 2.4.6. Bile Salt Hydrolase (BSH) Activity

The probiotic isolates were spotted on MRS agar plates supplemented with 0.5% of sodium taurodeoxycholic acid (Hi media, Mumbai, India) and 0.4% of calcium chloride. The plates were incubated at 37 °C for 72 h and observed for the precipitation zone around the growth [25].

### 2.5. Evaluation of LAB Isolates for Bacteriocin Production

The LAB isolates were assessed for their antimicrobial compounds using their neutralized CFS, CFS separately treated with catalase and trypsin. The CFS was obtained by centrifuging (at 10,000 rpm, 4 °C for 20 min) the broth cultures (24 h grown) and equally divided into three portions. One portion was adjusted to pH 7 with 10 M NaOH, the second part was treated with catalase (1 mg/mL) and the third part was added with trypsin (1 mg/mL) [26]. After 30 min of incubation, all the CFS was filter-sterilized (0.22 µm) and evaluated for their antimicrobial activity against *E. faecalis* MTCC439. The bacteriocin of the three isolates (BDUMBT08, 09, 11) was precipitated with ammonium salt (60%) and dialyzed overnight at 4 °C against sodium phosphate buffer (0.5 mM, pH 7.4) using Slide-A-Lyzer dialysis cassettes (3.5 k MWCO, Thermo Scientific, Rockford, IL, USA). The dialysate was used to confirm the presence of bacteriocin and its molecular weight by SDS-PAGE (15% gel). The molecular weight of the protein fraction was determined by comparing it with standard protein markers (11−250 kDa, New England Biolabs, Ipswich, MA, USA).

### 2.6. Safety Evaluation of Probiotic LAB

#### 2.6.1. Hemolysin Activity

Bacterial culture (24 h old) was streaked as a single line on blood agar plates (5% defibrinated blood) and incubated at 37 °C for 72 h. The plates were checked for hemolytic reaction by observing the clear zone of lysis around the growth (β-hemolysis), incomplete or partial lysis with the green zone (α-hemolysis), and no zone (γ-hemolysis).

#### 2.6.2. Biofilm Production

The potential of isolates to produce biofilm was checked by the glass tube method. About 10 µL of log-phase culture was inoculated into a glass tube containing 1 mL of sterile MRS broth and the tubes were incubated statically at 37 °C for 48 h. The broth culture was discarded and the tubes were rinsed thrice with PBS to remove the adhered cells. One mL of crystal violet (0.1%) was added to all the tubes and incubated for 30 min at room temperature. The stain was discarded and tubes were washed twice with PBS and air dried and observed for blue colour revealing the biofilm formation. The positive control tube was maintained with biofilm positive culture.

#### 2.6.3. Antibiotic Sensitivity Pattern

Disc diffusion assay was demonstrated to determine antibiotic sensitivity pattern of probiotic isolates using the antibiotics namely, cell wall inhibitors: ampicillin cephalosporin, carbapenems, and vancomycin; nucleic acid synthesis inhibitors: ciprofloxacin, norfloxacin, and rifampicin; protein synthesis inhibitors: chloramphenicol, erythromycin, gentamicin, and streptomycin; and antifolate antibiotics: trimethoprim and sulfamethoxazole. About 50 µL culture suspension containing 2.4 × 10^8^ CFU/mL was swabbed on the surface of Mueller Hinton agar (MHA) plates and antibiotic discs were placed after being allowed to absorb excess moisture. All the plates were incubated at 37 °C for 24 h. The antibiotic sensitivity/resistance pattern of the LAB isolates was determined by following CLSI 2017 guidelines.

### 2.7. Genotypic Identification of Probiotic LAB

The genomic DNA from LAB isolates was extracted using QIAamp DNA mini kit (Qiagen, Germantown, MD, USA) by following the manufacturer’s instruction and 16S rRNA gene was amplified using universal primers (details given in Supplementary File Table S1). The purified PCR product was submitted for sequencing.

The 16S rRNA gene sequences from seven LAB isolates were analyzed using the Basic Local Alignment Search Tool (BLAST) of National Centre of Biotechnology Information (NCBI) to determine the sequence similarity. Furthermore, phylogenetic tree was constructed using MEGA version 10 after the sequences were aligned with the CLUSTAL W program. Bootstrapping was also carried out with 1000 replicates and the evolutionary relationship of breast milk isolates was determined by neighbor joining method.

### 2.8. Statistical Analysis

All the data from three independent experiments were analyzed in Prism (GraphPad version 6) using ANOVA. A level of difference at *p* ≥ 0.05 was considered statistically significant.

## 3. Result

**Figure 1:**
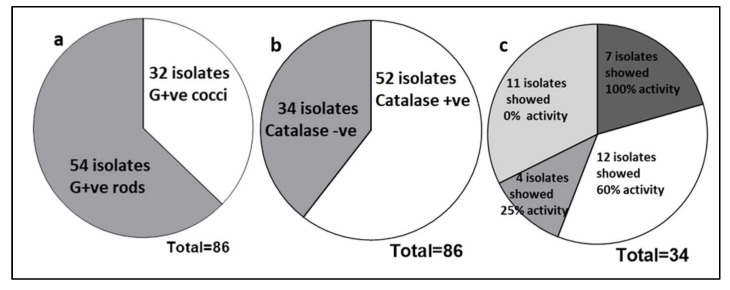
Breast milk bacterial profile based on their (**a**) Gram’s reaction, (**b**) Catalase test, and (**c**) Antimicrobial spectrum against indicator strains (12 nos.).

**Figure 2:**
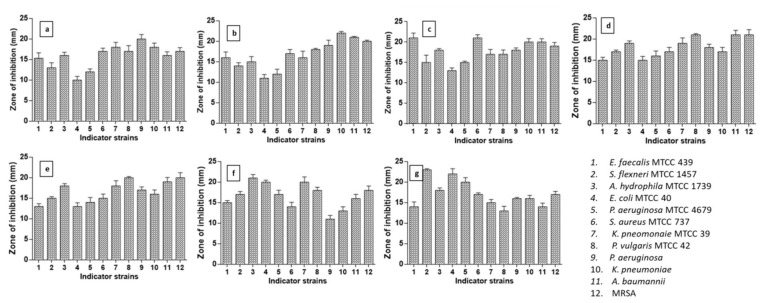
Antimicrobial spectrum of (**a**) BDUMBT08, (**b**) BDUMBT09, (**c**) BDUMBT10, (**d**) BDUMBT11, (**e**) BDUMBT12, (**f**) BDUMBT13, and (**g**) M2403 isolated from breast milk against indicator strains (MTCC strains and clinical pathogens) after 15 h incubation at 37 °C.

A total of 86 bacterial strains were isolated from milk samples of eight lactating mothers (Supplementary File Table S2). Amongst, 54 isolates were found to be Gram positive rods and the remaining 32 isolates were Gram-positive cocci (Figure 1a). About 34 Gram-positive and catalase and oxidase negative isolates (Figure 1b) were further evaluated for antimicrobial spectrum, out of that, seven isolates showed 100% antibacterial activity (Figure 1c); nevertheless, the degrees of the antimicrobial pattern is varied from each isolate as evident from the zone of inhibition (Figure 2). Sixty percent of activity was observed in 12 bacterial isolates and 25% of activity was observed in four isolates. Eleven isolates showed no activity against any pathogens (Figure 1c).

The results of antagonistic activity of LAB showed that all the LAB, except BDUMBT08, exhibited strong antagonism (≤20 mm zone of inhibition) against nearly 25% of indicators and all they showed good activity (zone of inhibition of 15–20 mm) against 75% of indicators. Furthermore, the results also revealed that all the isolated strains, except BDUMBT13, showed good antagonism (15–21 mm zone of inhibition) against clinical pathogens (*P. aeruginosa*, *K. pneumoniae*, *A. boumannii*, and MRSA) (Figure 2). The morphological and phenotypic characteristics of the seven isolates selected for further study were given in Table 1.

### 3.1. Assessment of Probiotic Potential

#### Evaluation of Gastrointestinal Survival

**Figure 3:**
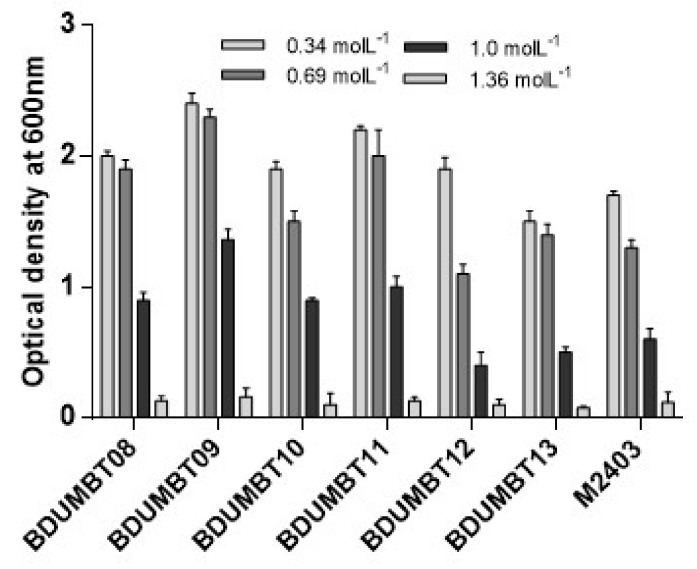
Tolerance of LAB isolates to different concentrations of NaCl (0.34, 0.69, 1.0, and 1.36 mol/L). The survival was monitored by reading the OD of the broth culture at 600 nm after 24 h incubation at 37 °C.

Breast milk LAB isolates were screened for their viability at lysozyme (100 µg/mL), acidic (pH 2–4), and bile salt (0.5% and 1%) for 3 h incubation. Table 2 depicted the growth of various LAB isolates under various conditions. All the 7 LAB isolates showed higher resistance to lysozyme with an average survival rate of 77% after 3 h exposure. The isolate BDUMBT09 showed highest lysozyme resistance (81%) after 3 h exposure and was followed by BDUMBT12 (80%) and BDUMBT13 (79%). The least tolerance was observed in BDUMBT08 with 70%. In acid tolerance assay, BDUMBT09 and BDUMBT08 were highly resistant (64% and 63% respectively) at pH 2 after 3 h incubation. BDUMBT08, BDUMBT09, and BDUMBT12 showed nearly 80% survival rate at pH 3 after 3 h exposure. For all LAB strains a less reduction of CFU (1–2 log CFU) was observed at pH 4 when compared to the control (pH 7) (Table 2). Similarly, majority of the isolates (BDUMBT08, BDUMBT09, BDUMBT10, and BDUMBT11) showed high viability (>70%) at 0.5% bile after 3 h exposure. However, a significant reduction in viability was observed for all the isolates at increased bile concentration (1%) (Table 2). The viability of 7 LAB in artificial gastric fluid (AGF) at pH 2.5 and artificial intestinal fluid (AIF) at pH 8.0 was evaluated, which helps in inhabiting the GIT. All the strains showed 50% of viability at pH 2.5 and the highest viability of 55% in AGF was observed in BDUMBT09 and BDUMBT10, and the least survival of 50% was observed in BDUMBT12. However, all the isolates showed more than 60% viability in AIF (pH 8). Highest and least survival was observed in BDUMBT08 (75%) and BDUMBT10 (60%) (Table 3). The osmo-tolerance of the isolates was checked by growing them in various concentrations of NaCl (0.34, 0.69, 1.0, and 1.36 mol/L). The highest absorbance (2.34) was observed in BDUMBT09 at 0.69 mol/L of NaCl and it was reduced to 1.36 at 1 mol/L. Similarly, the least absorbance (1.13) was observed in BDUMBT12 at 0.69 mol/L of NaCl (Figure 3) and showing their faint tolerance to high concentration of NaCl. The entire tested LAB showed least tolerance at 1.0 mol/L and very least tolerance at 1.36 mol/L. In this study, seven LAB were assessed for BSH activity and four LAB—namely, BDUMBT08, BDUMB09, BDUMBT10, and BDUMBT11—produced precipitation zone around their growth (after 72 h) showing their potential to withstand the presence of conjugated bile in the duodenum.

### 3.2. Evaluation of Cell Surface Properties

**Figure 4:**
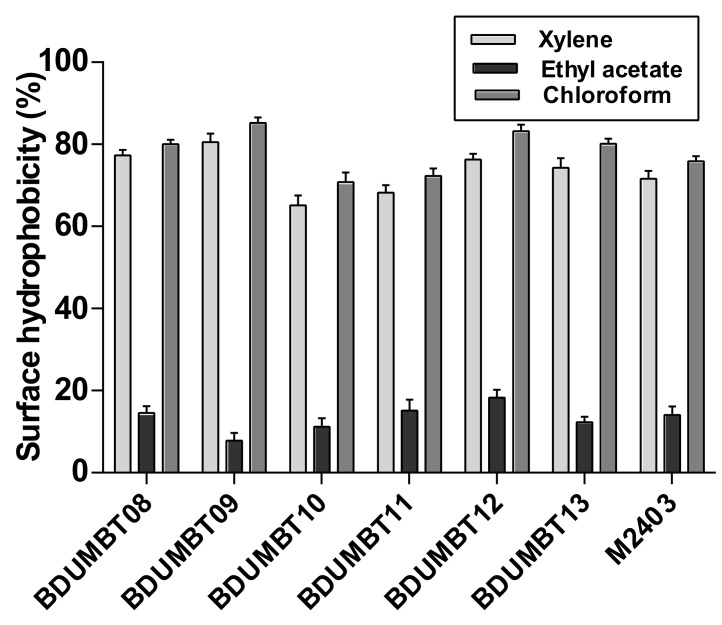
Cell surface hydrophobicity of LAB isolates as determined in three different hydrocarbons, xylene, ethyl acetate, and chloroform after 1 h incubation at 37 °C. Data used to plot are mean with SD from triplicate experiments.

The Figure 4 described the adherence of the bacterial isolates to xylene, chloroform, and ethyl acetate. For chloroform, the highest hydrophobicity was observed in BDUMBT09 (85%) and followed by BDUMBT12 (83%), BDUMBT08 (80%), and BDUMBT13 (80%). A distinct result was observed for ethyl acetate and the affinity was ranged from 8–18%.

**Figure 5:**
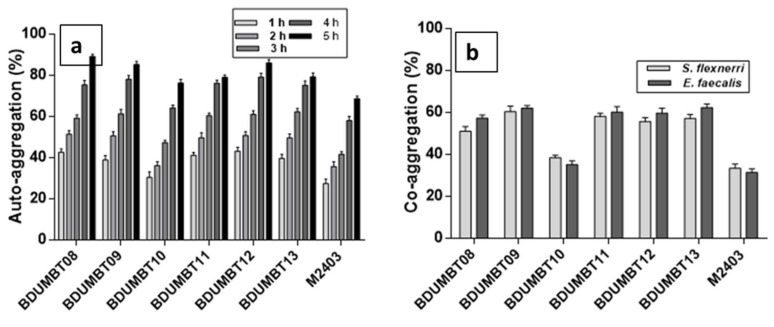
(**a**) Auto-aggregation of LAB isolates at various time points (after 1, 2, 3, 4, and 5 h) in PBS; (**b**) Co-aggregation of LAB with two different pathogens *S. flexneri* and *E. faecalis* after 5 h incubation in PBS. Data shown are mean with SD from three independent experiments.

**Figure 6:**
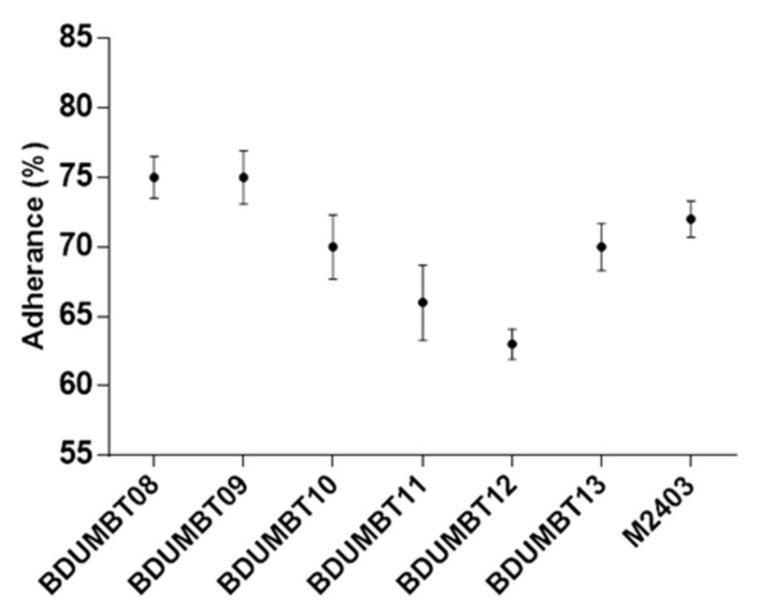
Mucin adherence assay. Adherence percentage is calculated by enumerating the CFU of LAB before and after adherence to mucin. Data plotted are mean with SD from three independent experiments.

Among the seven isolates, five isolates (excepting BDUMBT10 and M2403) showed more than 50% of aggregation within 2 h and further incubation increased their aggregation potential up to 89% (Figure 5a). The co-aggregation potential of the seven isolates was shown in Figure 5b. All the isolates showed better co-aggregation with *E. faecalis* than *S. flexneri*. A high co-aggregation percentage (62%) was recorded for BDUMBT09 and BDUMBT13 with *E. faecalis* and was followed by BDUMBT11 and BDUMBT12 with 60%. Figure 6 described the mucin adherence of the LAB isolates and it is in the range of 63–75%. The isolates, BDUMBT08 and BDUMBT09 showed the highest adherence to mucin (75%) and followed by M2403 with 72%. The remaining isolates showed more than 60% adherence to mucin.

### 3.3. Characterization of Antimicrobial Compounds

**Figure 7:**
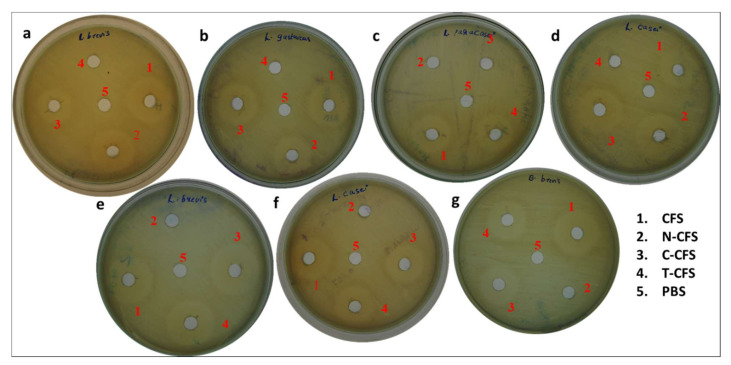
Antimicrobial activity of CFS, neutralized CFS (N-CFS), catalase treated CFS (C-CFS) and trypsin treated CFS (T-CFS) of (**a**) BDUMBT08, (**b**) BDUMBT09, (**c**) BDUMBT10, (**d**) BDUMBT11, (**e**) BDUMBT12, (**f**) BDUMBT13, and (**g**) M2403 isolated from breast milk against *E. faecalis* 439 after 15 h incubation at 37 °C.

**Figure 8:**
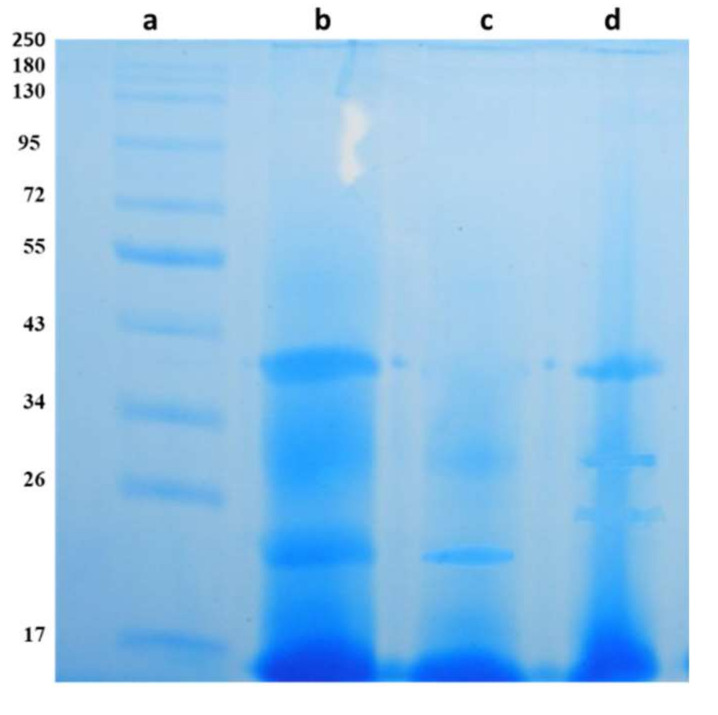
SDS-PAGE analysis of crude bacteriocin (dialysed sample) in 15% of gel stained with Coomassie brilliant blue R-250. Lane a: NEB Blue prestained protein standard (11–250 kDa); Lane b: crude sample from BDUMBT08; Lane c: BDUMBT09; Lane d: BDUMBT11.

The ability of probiotic LAB to produce bacteriocin was analyzed using various forms of CFS, CFS, neutralized CFS (N-CFS), catalase treated CFS (C-CFS), and trypsin treated CFS (T-CFS). A zone of inhibition against *E. faecalis* was observed in wells added with CFS, C-CFS, and T-CFS from isolates BDUMBT12, BDUMBT13 and M2403, whereas BDUMBT08, BDUMBT09, and BDUMBT11 produced different patterns of the zone of inhibition, their CFS, N-CFS, and C-CFS only produced antimicrobial activity against indicator strain. BDUMBT10 produced a zone of inhibition only by CFS and T-CFS (Figure 7). The SDS-PAGE analysis of the crude sample revealed a band of molecular weight of 40 kDa for BDUMBT08 and BDUMBT11, and 22 kDa for BDUMBT09 (Figure 8).

### 3.4. Biosafety Assessment of Probiotic LAB

All the seven isolates were assessed for their safety by analyzing their ability to lyse blood cells, to form biofilm and their antibiotic sensitivity profile as these are considered as major virulence factors. All the isolates did not produce any type of zone around their growth indicating their non-hemolytic nature. Similarly, they were negative for biofilm formation as biofilm formation was not observed in test tubes. Furthermore, the LAB was checked for sensitivity pattern to four various groups of antibiotics as described earlier (13 antibiotics). The results showed that BDUMBT08 and BDUMBT11 were sensitive to all the antibiotics tested and BDUMBT09 was found to be resistant to chloramphenicol, erythromycin, and streptomycin. The remaining four isolates—namely, BDUMBT11, BDUMBT12, BDUMBT13, and M2403—were resistant to erythromycin and streptomycin, and sensitive to all other antibiotics (Table 4).

### 3.5. Genotypic Analysis of Probiotic LAB

**Figure 9:**
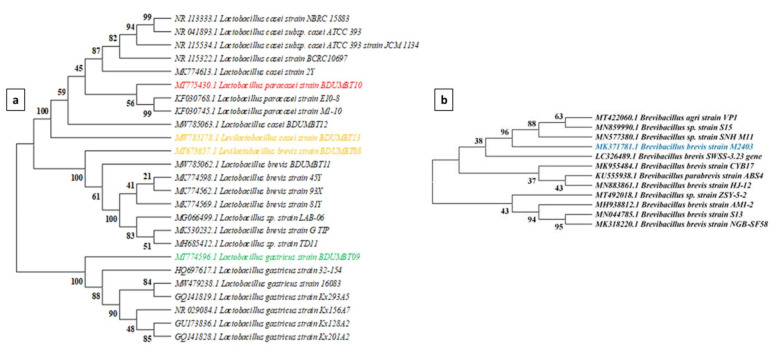
Phylogenetic tree constructed using 16S rRNA gene sequences of (**a**) seven *Lactobacillus* strains and 19 sequences retrieved from GenBank (**b**) *Brevibacillus brevis*. Except a few nodes, all have greater than 70 bootstrap values (>50) and noted at the node of the tree indicating the maximum likelihood of the tree.

The 16S rRNA gene sequences obtained from PCR amplified products of 7 LAB were analyzed in BLASTN and showed that among the seven isolates, M2403 showed 100% similarity to *Brevibacillus brevis*. Other strains namely, BDUMBT08 and BDUMBT11 are matched with *L. brevis* (97 and 98%), BDUMBT09 with *L. gastricus* (97%), BDUMBT10 with *L. paracasei* (97%) and BDUMBT12 and BDUMBT13 with *L. casei* (99 and 98%). The expect value (E value) of the BLASTN analysis of all the isolates were found to be 0. The 16S rRNA gene sequences of seven LAB were submitted to GenBank and their accession numbers with their E value were listed in Table 5. The phylogenetic tree for 6 various *Lactobacillus* species (Figure 9a) was constructed based on 16S rRNA gene sequences analysis and described the lineal relationship among the six *Lactobacillus* strains and 19 strains retrieved from the GenBank. Similarly, the tree was constructed for *B. brevis* M2403 sequences (Figure 9b) against 11 strains from GenBank.

## 4. Discussion

Breast milk is an exclusive food of newborns at least up to their 6 months due to the presence of complete both nutritional and non-nutritional bioactives. Furthermore, it is the most significant source of healthy microbiota of infants’ gut and provides gastrointestinal homeostasis [27]. Metagenomic analysis of breast milk revealed the important perceptions into microbial flora that are associated with intestinal maturation [28]. An exploratory study on the screening of probiotic LAB from breast milk based on their safety attributes was conducted herein. The spectrum of antimicrobial activity is varied from strains, and all the seven isolates evaluated in this study showed antimicrobial activity against all the tested indicator pathogens. The wide spectrum of antimicrobial activity of probiotic LAB against infectious pathogens has directed them to be the better alternatives to treat various infectious diseases. The antimicrobial activity is mainly due to the production of organic acids, diacetyl, acetoin, hydrogen peroxide, and bacteriocins [29,30].

### 4.1. Gastrointestinal Tolerance

The buccal cavity is the first challenging area for probiotics to withstand the high concentration of lysozyme in saliva [31] and is followed by harsh conditions of the GIT. Thus, the probiotics must be survived during the passage through the upper GIT, especially the highly acidic stomach and bile nature of the small intestine [32]. The survival of LAB under the simulated GIT conditions was tested for 3 h as the time of residing the bacteria in the stomach is to be approximately 90 min [33]. All the seven LAB isolates showed an acceptable level of tolerance to lysozyme as the least tolerance (70%) showed by BDUMBT08 is comparable to other previously reported strains, *L. palntarum* (69%), *L. fermentum* CECT5716 (72%), and *W. paramesenteroides* (80%) [34]. These variations are mainly due to the physiological state of the cell and composition of the cell walls, especially the peptidoglycan layer because they are mainly attributed to the potent of lysozyme tolerance [34].

In this study, an acid tolerance assay was carried out at low pH 2, 3, and 4 as the antimicrobicidal activity of the stomach acid is to be at pH 2.5 [35]. The present results showed that the survival rate of all tested LAB at pH 2 was less when compared to pH 3 and 4. The probiotics such as *L. plantarum* [36] and *Bifidobacterium* strains [37] were much more resistant to the acidic conditions at pH 3 than at pH 2. The probiotics should possess high intestinal deliverability in order to exert their benefits. Thus, the resistance to stomach acid, bile, and gastric juice is imperative to select a probiotic strain [38]. According to the patent numbered US 2016/015.1434 A1, the probiotics *L. plantarum* strains APsulloc 331261, 331263, and 331266 have excellent acid resistance specifically at pH 2.5–3.5 for 3 h and showed 0% viability at pH 2. The current strains (evaluated in the present study) have 50% viability at pH 2 after 3 h exposure and showed their excellence of survival in extreme acidic conditions (pH 2), resembling the empty stomach pH about 1.2–2.0. Thus, the current probiotics may have high intestinal delivery when consumed.

At 0.5% of bile, all the isolates showed more than 65% viability. At the same time, the highest reduction (more than 4 log CFU) was observed for all the isolates when they exposed to 1% bile and this is due to the damage of the bacterial cell membrane consisting of lipids and fatty acids. Furthermore, the bile resistance was mostly related to deconjugating the bile salt, which will help in protecting the normal flora of the gut region [39]. The tolerance of LAB strains to AGF and AIF was conducted and revealed that seven LAB isolates could sustain the artificial gastric and pancreatic digestive conditions with minimal loss of viable cells after 3 h, which could be sufficient to withstand and reach their destination in the intestine to exert beneficial activities. The current isolates showed a higher survival rate under simulated gastric juice than *L*. *rhamnosus* GG (54.9%) [16] indicating their high potential to grow under simulated gastric conditions.

The osmo-tolerance is one of the preferred features of probiotics since they must survive in the GIT where the osmolarity is equivalent to 0.3 mol/L NaCl [22]. The tested isolates showed a varying level of tolerance at each concentration of NaCl indicating the osmo-tolerance is strain specific. Except for BDUMBT12, BDUMBT13, and M2403, the remaining four isolates showed good tolerance up to 0.6 mol/L of NaCl. The present results are comparably higher than the previously reported *L. fermentum* strains from different curd samples showed the highest tolerance up to 0.34 mol/L and lowest tolerance at 1.0 mol/L in NaCl [40].

The world health organization (WHO) recently announced bile salt deconjugation as one of the significant features of bacteria for considering as probiotics [5] seemed to be related to reduction in blood cholesterol level [41]. Above 50% of the isolates proved their deconjugation ability by forming precipitation zone around their growth and all these isolates already showed their very good bile tolerance at 0.5% bile. This deconjugation property is commonly observed in *Lactobacillus*, *Enterococcus*, and *Bifidobacterium* from the human GIT [42]. Similarly, hydrophobicity is one of the significant factors mediating the adherence of probiotics to the host gut and non-specific adherence [43] and this could help to maintain and colonize the human GIT [29]. Moreover, this test also reveals the hydrophobic nature of the cell surface. Similarly, a high affinity to chloroform (polar acidic solvent) and ethyl acetate (polar basic solvent) describes the electron donor and acceptor potential of bacterial cell surface respectively [44], which is endorsed to carboxylic groups and acid–base interactions [23]. The present results described that the affinity to chloroform is significantly high among all the tested isolates indicating their electron donor potential. Similarly, a high percentage of bacterial adherence towards xylene reveals the hydrophobic nature of the cell surface. An earlier report on physicochemical studies illustrated that the proteinaceous materials of bacterial cell surfaces contribute high hydrophobic nature to the cell [23].

Auto-aggregation plays a role in the adhesion and colonization ability of probiotics on the host intestinal epithelial cells [45]. According to the current results, all the isolates exhibited a good aggregation phenotype which ranged from 69% to 89% after 5 h incubation. Likewise, co-aggregation potential allows the probiotic cells to form a barrier that prevents pathogenic colonization by creating an environment with a high concentration of inhibitory compounds [46]. From the reports of Gil et al. [47], Al Kassaa et al. [48], Santos et al. [46], the current results showed different levels of auto and co-aggregation among the different species, proposing that these abilities depend on the strains. Furthermore, most probably, aggregation potential is related to the cell adhesion property [49] and this hypothesis was also perceived in our study, as the isolates demonstrating stronger aggregation and co-aggregation potential showed higher adherence to polar acidic solvent, chloroform.

The mucoadhesion of LAB was investigated in this study as the mucin layer present on the GIT [50] acts as a barrier to protect the epithelium from pathogenic adhesion. In mucoadhesion assay, type III porcine gastric mucin was used as a matrix to evaluate its adhesion capacity in vitro, since it is regularly used as a model for humans in bacterial adherence studies [51,52]. Herein, all the isolates exhibited good mucoadhesion in the range of 63–75% revealing their ability to successfully colonize on the intestinal mucosa. Earlier studies showed that 70% and 65% mucin adherence was observed for *L. plantarum* strains [50] and *L. mucosae* LM1 [53] respectively.

### 4.2. Bacteriocinogenic Efficacy of LAB Isolates

LAB with antimicrobial activity is known to produce many antimicrobial compounds such as various organic acids, H_2_O_2_, ethanol, and bacteriocin. The isolates with a broad spectrum of antimicrobial activity were checked for their ability to produce bacteriocin-like substances using their CFS. The results revealed that CFS of all the isolates showed antimicrobial activity and their treated CFS like N-CFS, C-CFS, and T-CFS varied in their antimicrobial activity. The C-CFS and T-CFS of isolates, BDUMBT12, BDUMBT13, and M2403 showed antimicrobial activity against the indicator strain revealing their inhibitory activity is due to organic acids and not due to H_2_O_2_ and bacteriocin. The N-CFS and C-CFS of the remaining three LAB—namely, BDUMBT08, BDUMBT09, and BDUMBT11—produced inhibition zone against the indicator strain; apparently representing the activity is not due to H_2_O_2_ and any other acids since the activity of organic acids and H_2_O_2_ was excluded by neutralizing the solution and treating with catalase respectively. Furthermore, the proteinaceous nature of the antimicrobial compound of BDUMBT08, BDUMBT09, and BDUMBT11 is confirmed as the compound completely lost its activity after treating with proteolytic enzymes. SDS-PAGE also confirmed the presence of bacteriocin and its molecular mass. In addition, it is needed to be purified before other functional analyses. The previous reports of Banerjee et al. [54], Kumari et al. [55], and Gao et al. [56] revealed that the bacteriocin of various strains of Lactobacillus differ in their molecular mass and characteristics. Bacteriocin producing *Pediococcus acidilactis* NMCC11 was isolated from buffalo milk and their antimicrobial activity was reported against clinical pathogens [57]. The bacteriocinogenic potential of the three LAB (BDUMBT08, BDUMBT09, and BDUMBT11) may be an added advantage of probiotics, as they help the producer strain for their survival in GIT and inhibit the growth of the pathogens by acting as signaling peptides in the gut environment.

### 4.3. Biosafety Assessment of Probiotic LAB

According to FAO regulations, bacterial strains to be considered as probiotics must be safe to the host. Thus, the probiotic strains must be devoid of hemolytic activity, biofilm-producing ability, and antimicrobial resistance as these are considered virulence determinants. In this study, all these assays were chosen based on the international guidelines for evaluating the potent probiotics [5]. The seven LABs were enrolled for safety assessment and confirmed their safety, since all these strains showed gamma type of hemolysis. Besides, all the seven isolates did not produce biofilm. The isolates showing negative hemolytic activity could not be converted to opportunistic pathogens [58]. Previous studies [12,37,59] also confirmed that the majority of LAB isolates showed negative for hemolysis assay.

According to European Food Safety Authority (EFSA), the assessment of susceptibility of probiotics to antibiotics is also one of the prerequisites in screening probiotics. EFSA recommended using any two groups of antibiotics (cell wall inhibitors, ampicillin and vancomycin and protein inhibitors, chloramphenicol, gentamicin, clindamycin, erythromycin, streptomycin, kanamycin, and tetracycline) for proper screening of functional probiotic strains. Herein, BDUMBT08 and BDUMBT11 are completely sensitive to all the antibiotics used in this study and all others are resistant to 2–3 antibiotics—namely, chloramphenicol, erythromycin and streptomycin. Resistance to aminoglycosides could be an intrinsic feature among LAB [59]. Erythromycin and clindamycin-resistant *Bifidobacterium longum* IPLA 20001 from breast milk was reported by Arboleya et al. [60]. Similarly, tetracycline and streptomycin-resistant LAB from neera was reported by Somashekaraiah et al. [12]. These studies suggested that further clarification is necessary to study the genetic basis and ability to transfer these resistance genes. The strains with non-transferrable resistance to few antibiotics will be useful during the condition of co-administration with antibiotics [60].

### 4.4. Identification Probiotic LAB

From the BLASTN analysis, the reasonable similarities (97%) found in all the LAB isolates confirmed the bacterial identity with their respective reference strain, since the sequences with ≥97% similarity with reference sequences could be considered as criteria for species identification [26]. Furthermore, the E (Expect) value of BLASTN analysis of all the seven isolates is found to be 0.0 indicating high similarity between a query and reference sequences, since the smaller the E value (closer to 0) the more similarity of bacterial sequence results to the one being matched (available online: https://blast.ncbi.nlm.nih.gov (accessed on 30 December 2021)).

## 5. Conclusions

The screening and characterization of LAB from breast milk towards identifying potent probiotics are the major objectives of this study. Although a large number of probiotics have been reported from human breast milk, characterization of new probiotic strains is always valuable as each strain exhibits different probiotic traits with several health benefits. In this study—based on various physiological, biosafety, and probiotic characterization—the seven isolates such as *L. brevis* BDUMBT08 and BDUMBT11; *L. gastricus* BDUMBT09; *L. paracasei* BDUMBT10; *L. casei* BDUMBT12 and BDUMBT13; and *B. brevis* M2403 are identified as potent probiotic strains. Their strong antimicrobial activity can inhibit the adhesion of pathogens to the intestinal epithelial cells. Their resistance to gastrointestinal conditions, surface binding, auto- and co-aggregation, and mucoadhesion enable them to colonize on the GIT which in turn helps them to strengthen the intestinal barrier functions and promote the defense against infectious diseases. Additionally, bacteriocin-producing facet of *L. brevis* BDUMBT08 and BDUMBT11 and *L. gastricus* BDUMBT09 could be evidenced for their biopreservative efficacy along with their therapeutic role. The overall results concluded that breast milk is a potential source of probiotics for infants’ gut microbiome. Since all seven LAB proved their probiotic and safety efficacy with strong antimicrobial activity against human pathogens, they could be used as a therapeutic agent to treat infectious diseases. Thus, the human-originated LAB strains are being furthermore characterized to exploit their probiotic and therapeutic potential towards specific human welfare in the form of customized functional foods and infant formulas.

## Figures and Tables

**Table 1:** Phenotypic (morphological and physiological) analysis, fermentation ability and growth at different temperature of various bacterial isolates.

Tests	Breast Milk Bacterial Isolates						
	BDUMBT08	BDUMBT09	BDUMBT10	BDUMBT11	BDUMBT12	BDUMBT13	M2403
**Morphological**							
Gram’s staining	+ve rods	+ve rods	+ve rods	+ve rods	+ve rods	+ve rods	+ve rods
Endospore production	–	–	–	–	–	–	–
Motility	–	–	–	–	–	–	–
**Physiological**							
Catalase production	–	–	–	–	–	–	–
Oxidase production	–	–	–	–	–	–	+
Indole production	–	–	–	–	–	–	–
Acid production	–	–	–	–	–	–	–
Acetoin production	–	–	–	–	–	–	–
Citrate hydrolysis	–	–	–	–	–	–	–
Urease production	–	–	–	–	–	–	–
Starch hydrolysis	–	–	–	–	–	–	+
**Carbohydrate fermentation**							
Lactose	+	+	+	+	+	+	+
Glucose	+	+	+	+	+	+	+
Mannitol	+	–	+	+	+	+	–
Fructose	+	+	+	+	+	+	+
Sorbitol	+	+	+	+	+	+	+
Maltose	+	+	+	+	+	+	+
Galactose	+	+	+	+	+	+	+
Mannose	+	+	–	+	+	+	+
Arabinose	–	–	–	–	–	–	–
**Growth at different temperature**							
4	–	–	–	–	–	–	–
25	++	++	++	++			++
37	+++	+++	+++	+++			+++
45	–	–	–	–	–	–	+

+: positive, –: negative, ++: growth, +++: well growth.

**Table 2:** Survival rate of LAB isolates after 3 h exposure to lysozyme (100 µg), acidic (pH 2, 3, 4) and bile (0.5%, 1%) conditions and expressed in log CFU and percentage of survival. Each experiment is individually maintained with separate control.

Bacterial Isolates	Tolerance to Lysozyme			Tolerance to Acid							Tolerance to Bile				
				At pH 2		At pH 3		At pH 4		Control	At 0.5%		At 1%		Control
	Log CFU	S (%)	Control	Log CFU	S (%)	Log CFU	S (%)	Log CFU	S (%)	Log CFU	Log CFU	S (%)	Log CFU	S (%)	Log CFU
BDUMBT08	6.7 ± 0.16	70	9.6 ± 0.7	5.7 ± 0.13	63	7.1 ± 0.13	78	8.5 ± 0.13	93	9.1 ± 0.03	6.8 ± 0.18	71	5.2 ± 0.16	54	9.6 ± 0.7
BDUMBT09	7.7 ± 0.18	81	9.5 ± 1.5	5.9 ± 0.18	64	7.4 ± 0.23	80	8.6 ± 0.08	94	9.2 ± 0.08	7.0 ± 0.13	71	5.0 ± 0.13	51	9.9 ± 1.5
BDUMBT10	7.4 ± 0.13	78	9.5 ± 1.9	5.2 ± 0.19	58	6.9 ± 0.35	77	8.2 ± 0.11	91	9.0 ± 0.16	7.1 ± 0.17	75	5.6 ± 1.0	59	9.5 ± 1.9
BDUMBT11	6.8 ± 0.34	72	9.5 ± 0.7	5.6 ± 0.09	59	7.2 ± 0.31	76	8.4 ± 0.16	88	9.5 ± 0.16	6.9 ± 0.23	71	4.8 ± 0.5	50	9.7 ± 0.7
BDUMBT12	7.6 ± 0.38	80	9.5 ± 0.4	5.1 ± 0.16	55	7.4 ± 0.13	80	8.3 ± 0.18	89	9.3 ± 0.18	6.3 ± 0.21	67	4.4 ± 0.8	47	9.4 ± 0.4
BDUMBT13	7.4 ± 0.21	79	9.4 ± 0.13	5.0 ± 0.13	53	7.2 ± 0.41	76	7.8 ± 0.13	82	9.5 ± 0.13	6.5 ± 0.13	68	4.9 ± 1.3	52	9.5 ± 0.31
M2403	7.0 ± 0.27	74	9.5 ± 0.18	4.5 ± 0.18	49	6.1 ± 0.17	66	7.3 ± 0.16	79	9.2 ± 0.0	6.7 ± 0.2	67	4.7 ± 0.14	50	9.5 ± 1.9

**Table 3:** Effect of AGF (pH 2.5) and AIF (pH 8) on the survival rate of LAB isolates after 3 h.

Bacterial Isolates	Tolerance to AGF		Tolerance to AIF		Control
	Log CFU	S (%)	Log CFU	S (%)	
BDUMBT08	5.0 ± 0.06	52	7.2 ± 0.13	75	9.6 ± 0.7
BDUMBT09	5.4 ± 0.18	55	7.0 ± 0.02	71	9.9 ± 1.5
BDUMBT10	5.2 ± 0.12	55	6.0 ± 0.58	60	9.5 ± 1.9
BDUMBT11	6.0 ± 0.15	51	7.1 ± 0.09	73	9.7 ± 0.7
BDUMBT12	4.7 ± 0.08	50	5.9 ± 0.08	63	9.4 ± 0.4
BDUMBT13	5.0 ± 0.07	53	6.9 ± 0.15	67	9.5 ± 0.13
M2403	4.8 ± 0.04	51	6.3 ± 0.15	66	9.5 ± 1.9

**Table 4:** Summary of hemolysis (in 5% blood agar) assay, biofilm production, and antibiotic susceptibility of LAB isolates.

Breast Milk Isolates	Hemolysis Activity	Biofilm Production	Zone of Inhibition (mm) and Score * as Resistant and Sensitive												
			AMP	CEP	Car	VAN	CIP	NOR	RIF	C	ERY	GEN	STREP	TRI	SX
BDUMBT08	–	–	23 S	24 S	20 S	21 S	18 S	20 S	21 S	23 S	25 S	21 S	19 S	24 S	23 S
BDUMBT09	–	–	25 S	22 S	22 S	26 S	20 S	17 S	19 S	7R	0 R	18 S	8R	20 S	23 S
BDUMBT10	–	–	26 S	26 S	18 S	18 S	21 S	19 S	19 S	10 S	7 R	20 S	9 R	20 S	20 S
BDUMBT11	–	–	24 S	25 S	15 S	26 S	21 S	21 S	23 S	20 S	17 S	18 S	18 S	22 S	26 S
BDUMBT12	–	–	22 S	19 S	15 S	21 S	18 S	23 S	21 S	20 S	0 R	18 S	0 R	17 S	23 S
BDUMBT13	–	–	24 S	18 S	17 S	27 S	20 S	17 S	17 S	23 S	0 R	21 S	0 R	17 S	20 S
M2403	–	–	22 S	19 S	19 S	21 S	18 S	20 S	17 S	23 S	0 R	20 S	0 R	20 S	18 S

* The isolates were scored as resistant (R) andsensitive (S) based on the cut off values reported by Charteris et al. (1998). AMP: ampicillin; CEP: cephalosporin; CAR: carbapenems; VAN: vancomycin; CIP: ciprofloxacin; NOR: norfloxacin; RIF: rifampicin; C: chloramphenicol; ERY: erythromycin; GEN: gentamicin; STREP: streptomycin; TRI: trimethoprim; and SX: sulfamethoxazole. –: Negative; R: resistant; S: sensitive.

**Table 5:** Summary of BLASTN analysis of seven LAB isolates using their 16S rRNA gene sequences.

Breast Milk Bacterial Isolates	NCBI Accession No. of the Isolates	The Isolates Highly Matched with Species from Genbank	% of Query Coverage	E Value	% of Identity
BDUMBT08	MT673657	*Levilactobacillus brevis* strain BSO464	100	0	97
BDUMBT09	MT774596	*Lactobacillus gastricus* strain 32-154	100	0	97
BDUMBT10	MT775430	*Lactobacillus paracasei* strain Lp02	100	0	97
BDUMBT11	MW785062	*Levilactobacillus brevis* ATCC14869	100	0	98
BDUMBT12	MW785063	*Lactobacillus casei* subsp. *casei* ATCC393	99	0	99
BDUMBT13	MW785178	*Lactobacillus casei* strain BCRC10697	99	0	98
M2403	MK371781	*Brevibacillus brevis* strain HK544	99	0	100

## Data Availability

GenBank accession numbers for 16S rRNA sequences of isolated bacteria are MT673657, MT774596, MT775430, MW785062, MW785063, MW785178, and MK371781. All other data generated in this study are available from corresponding author on reasonable request.

## Supplementary Materials

The following are available online at https://www.mdpi.com/article/10.3390/cimb44020051/s1, Table S1: Details of primers and PCR conditions for amplifying 16S rRNA gene; Table S2: Screening of Lactic acid bacteria from the eighty six bacterial strains isolated from 8 lactating mothers breast milk.
