# Supplementary material for: Phenotypic Assessment of Probiotic and Bacteriocinogenic Efficacy of Indigenous LAB Strains from Human Breast Milk
Source: Curr Issues Mol Biol. 2022 Feb 1;44(2):731–49. doi: 10.3390/cimb44020051 (PMC8929004; doi:10.3390/cimb44020051)
Supplement: Supplementary file 1 [file cimb-44-00051-s001.zip › cimb-1558970-supplementary.pdf]

Table S1. Details of primers and PCR conditions for amplifying 16S rRNA gene

Primer name and its target gene	5’-3’ Sequences	PCR amplification conditions
27-F target 16S rRNA gene	AGAGTTTGATCCTGGCTCAG	Denaturation at 94 C for 5 min followed by 35 cycles of denaturation at 94 °C for 1 min; annealing at 56 °C for 1 S; extension at 72 °C for 3 min.
1492-R target 16S rRNA gene	GGT TACCTTGTTACGACTT	

Table S2. Screening of Lactic acid bacteria from the eighty six bacterial strains isolated from 8 lactating mothers breast milk

Bacterial isolates	Screening test			
	Gram’s staining	Endospore staining	Catalase test	Oxidase test
BDUMBT01	+ve rod	-ve	+ve	-ve
BDUMBT02	+ve rod	-ve	+ve	-ve
BDUMBT03	+ve cocci	-ve	-ve	+ve
BDUMBT04	+ve rod	-ve	+ve	-ve
BDUMBT05	+ve rod	-ve	+ve	-ve
BDUMBT06	+ve rod	-ve	+ve	-ve
BDUMBT07	+ve cocci	-ve	+ve	+ve
**BDUMBT08**	**+ve rod**	**-ve**	**-ve**	**-ve**
**BDUMBT09**	**+ve rod**	**-ve**	**-ve**	**-ve**
**BDUMBT10**	**+ve rod**	**-ve**	**-ve**	**-ve**
**BDUMBT11**	**+ve rod**	**-ve**	**-ve**	**-ve**
**BDUMBT12**	**+ve rod**	**-ve**	**-ve**	**-ve**
**BDUMBT13**	**+ve rod**	**-ve**	**-ve**	**-ve**
BDUMBT14	+ve cocci	-ve	+ve	+ve
BDUMBT15	+ve cocci	-ve	+ve	+ve
M2001	+ve rod	-ve	-ve	+ve
M2002	+ve rod	-ve	-ve	+ve
M2003	+ve rod	-ve	+ve	-ve
M2004	+ve rod	-ve	-ve	+ve
M2005	+ve rod	-ve	-ve	+ve
M2006	+ve cocci	-ve	+ve	-ve
M2007	+ve cocci	-ve	+ve	-ve
M2008	+ve cocci	-ve	+ve	-ve
M2009	+ve cocci	-ve	+ve	-ve
M2010	+ve rod	-ve	+ve	-ve
M2011	+ve cocci	-ve	+ve	-ve
M2012	+ve rod	-ve	+ve	-ve

M2013	+ve cocci	-ve	-ve	+ve
M2101	+ve rod	-ve	+ve	-ve
M2102	+ve cocci	-ve	+ve	-ve
M2103	+ve cocci	-ve	+ve	-ve
M2104	+ve rod	-ve	-ve	+ve
M2105	+ve cocci	-ve	+ve	-ve
M2106	+ve cocci	-ve	-ve	+ve
M2107	+ve rod	-ve	-ve	+ve
M2108	+ve cocci	-ve	-ve	+ve
M2109	+ve cocci	-ve	-ve	+ve
M2110	+ve cocci	-ve	+ve	+ve
M2111	+ve cocci	-ve	+ve	+ve
M2201	+ve rod	-ve	-ve	+ve
M2202	+ve cocci	-ve	-ve	+ve
M2203	+ve cocci	-ve	-ve	+ve
M2204	+ve cocci	-ve	+ve	-ve
M2205	+ve cocci	-ve	+ve	+ve
M2206	+ve rod	-ve	-ve	+ve
M2207	+ve rod	-ve	-ve	+ve
M2208	+ve rod	-ve	-ve	+ve
M2209	+ve rod	-ve	-ve	+ve
M2210	+ve cocci	-ve	+ve	-ve
M2211	+ve cocci	-ve	+ve	-ve
M2212	+ve rod	-ve	+ve	+ve
M2213	+ve cocci	-ve	+ve	-ve
M2301	+ve rod	-ve	+ve	+ve
M2302	+ve rod	-ve	+ve	+ve
M2303	+ve rod	-ve	-ve	+ve
M2304	+ve rod	-ve	+ve	+ve
M2305	+ve rod	-ve	+ve	+ve
M2306	+ve rod	-ve	+ve	+ve
M2307	+ve cocci	-ve	-ve	+ve
M2308	+ve cocci	-ve	-ve	+ve
M2309	+ve rod	-ve	-ve	+ve
M2310	+ve cocci	-ve	-ve	+ve
M2401	+ve cocci	-ve	+ve	-ve
M2402	+ve cocci	-ve	+ve	-ve
**M2403**	**+ve rod**	**-ve**	**-ve**	**-ve**
M2404	+ve cocci	-ve	+ve	-ve
M2405	+ve rod	-ve	+ve	-ve
M2406	+ve rod	-ve	-ve	+ve
M2407	+ve rod	-ve	+ve	-ve
M2408	+ve rod	-ve	+ve	-ve
M2409	+ve rod	-ve	-ve	+ve
M2501	+ve rod	-ve	+ve	-ve
M2502	+ve rod	-ve	-ve	+ve
M2503	+ve rod	-ve	+ve	-ve
M2504	+ve rod	-ve	+ve	-ve

M2505	+ve rod	-ve	+ve	+ve
M2506	+ve rod	-ve	+ve	-ve
M2507	+ve rod	-ve	+ve	-ve
M2508	+ve rod	-ve	+ve	-ve
M2601	+ve rod	-ve	+ve	-ve
M2602	+ve rod	-ve	+ve	-ve
M2603	+ve rod	-ve	+ve	+ve
M2604	+ve rod	-ve	+ve	-ve
M2605	+ve rod	-ve	+ve	+ve
M2606	+ve rod	-ve	+ve	-ve
M2607	+ve cocci	-ve	-ve	+ve
